# Supplementary material for: Gender bias in the Chilean public health system: Do we all wait the same?
Source: PLoS One. 2020 Sep 24;15(9):e0239445. doi: 10.1371/journal.pone.0239445 (PMC7515657; doi:10.1371/journal.pone.0239445)
Supplement: Attachment — Submitted filename: Response to reviewers.pdf [file pone.0239445.s002.pdf]

June 2, 2020

Editor PLOS ONE

Dear Academic Editor and Reviewers,

Thank you very much for the time you have devoted to reading our paper "Bias in the Chilean public health system: Do we all wait the same?" (PONE-D-20-03609) and for the insightful comments you have provided. The suggestions helped us to improve different aspects of the paper. We have created a new version of our manuscript in accordance with the comments of the reviewers. We notice that this new version has significantly changed, and therefore, we are not able to provide a friendly "readable" version highlighting all modifications.

We remark that after reviewer 1's comments, we revisited the calculations for waiting times. Thus, we slightly modified the number of opportunity guarantees considered in the study from 14 to 16, representing 15 diseases.

In what follows, we answer in detail the reviewer's comments.

Reviewer 1

1. *First of all, I think you should use more sophisticated methods to disentangle relationships involved. I think that regression-based analyses would be more appropriate in this case. The use of multiple regression would allow to account for the impact of various factors at the same time and test interactions between independent variables. What is more, because your observations are clustered (particular patients, types of provider, insurance status) I suggest that you consider multilevel approach to regression. Your results would be more robust than with the use of t-test.*

   We thank your thoughtful comment, and following your advice we have used a multilevel model, in addition to the Welch t-test. This substancial change in the paper, due to this new approach, is included in sections Materials and Methods, Results, and Discussion.

2. *I think you should explicitly state that you investigate gender bias in your title; it would be more informative.*

   Following your suggestion, we have included the gender bias in the title.

3. *Each time you use abbreviations first time in the text, they should be defined; this applies also to abstract (see GES, AUGE...).*

   Done it.

4. *The abstract itself provides insufficient details on your study. You do not*

mention the method used, the timespan of the analysis, the characteristics of the dataset. These critical details should be explicitly provided here.

Following your advice, we have rewritten the abstract using the following format: background, objective, methods, results and conclusion. We hope the new structure provides clearer details on our study.

5. *Both in abstract and conclusion you describe bias as 'unexpected'. Why are they unexpected? Several studies (you reference some of them) provide evidence on gender bias favoring men and thus your findings does not seem to be surprising.*

   The GES plan was introduced in order to level up differences in access and opportunities for the Chilean population. This is why we were not expecting to find such significant differences in waiting time between women and men. We have rewritten the Abstract and Conclusions to clarify this observation.

6. *The introductory part of your manuscript is a bit chaotic. The first and the last paragraphs of this part are concerned with Chilean health care and gender bias and between the two you review the previous literature on gender bias. I suggest that you reorganize it. Also, you start the introduction with explicit statement on the aim of your study. Usually, first some background information is provided, followed by brief literature review and this leads to formulation of knowledge gap. Here, some details which are typical for scientific introductions are missing, like the knowledge gap and explicit statement of your study's original contribution. Please reorganize this section according to more standard form.*

   Thank you for your comment. We have reorganized the Introduction according to your suggestion and believe it is much better now.

7. *Based on the referenced study [11] you state that '...healthcare coverage was twice as high for men as for women.' in Poland. According to the study [11] this statement applies to 'voluntary private insurance' which might be (and in fact it is here) very different than 'healthcare coverage'.*

   We have rewritten the citation. We apologize for this.

8. *First two paragraphs of the 'Overview of study design' are not something which could be labeled as materials and methods. This rather belongs to the content you now have in your very first paragraph of the introduction.*

   We have reorganized the introduction, including the first paragraphs you were referring to.

9. *More explanation is needed to describe why your approach selects only 14 of 80 diseases included in GES.*

After your comment we review how waiting time was computed with great detail. We finally included 16 GES–OG in the analysis corresponding to 15 diseases. In subsection Data (within section Materials and Methods), we have included the following paragraph explaining how waiting time is computed:

"We construct the waiting time (WT) as the time lapse between two interactions that have an explicit OG. It is worth noticing that not all diseases have the same guarantees, both in terms of maximum time allowed and also in the part of the care pathway that is covered. While for some diseases there are OGs for diagnostic confirmation, treatment and follow-up while, others include only treatment. Moreover, there are diseases in the GES plan for which the diagnostic confirmations do not generate an additional monetary transfer from FONASA to the provider that are not recorded in the database, preventing the construction of the WT (for instance the diagnostic confirmation of depression)."

10. *Lines 134-141 should rather be a footnote in the table than the main text which should describe findings instead.*
    We followed your suggestion and moved the columns' description to a footnote in the table. We have only included those that needed further explanation.

11. *More details on defining 'type of provider' variable would be beneficial.*
    We included the following paragraph in Data subsection with more details:

    "High complexity providers are larger hospitals (more than 300 beds) with 20% of its bed capacity assigned to critical care. They provide access to all (or most) subspecialties, diagnostic and treatment equipments. Medium complexity providers are hospitals with 31 to 300 beds, with a small proportion of beds for critical care, if any. They have lower level of infrastructure and equipments (no radiotherapy or chemotherapy, for instance) and they do not have all the subspecialties staffed. Low complexity providers are small hospitals with up to 30 beds, delivering only basic care. Health centers and health reference centers are medium-complexity institutions that provide only outpatient health services. However, they are responsible for referring patients within their health area who need care exceeding the center's level of complexity. In such cases, the institution pays for treatment and receives the FONASA reimbursement."

12. *Line 217: '... population in . ...'; it seems you have a typo.*
    This sentence is not longer part of the manuscript.

13. *Generally, the materials and methods and results sections would look different if you follow my comment on the use of regression-based analysis; therefore, I do not give more detailed recommendation on particular points therein.*
    Materials and Methods, Results and Discussion sections have substantially

changed.

14. *I like your discussion and the way you interpret your results; however, again, more could be done to make this section more standard in terms of usual content of discussion sections in scientific writing. I suggest to include general picture of your main findings at the beginning of discussion. But what is more important and in fact critical for decent discussion, you should definitely compare your results to previous studies from Chile and other settings. Moreover, you do not discuss limitations of your study at all while you should do so.*
We rewrote the Discussion section following your recommendations. We also mentioned the novelty of our study compared with the current literature on the subject. We included the following paragraph for limitations: "The main limitation of this study is that we did not explicitly consider the congestion of the healthcare system. Including the latter would be useful to understand compliance rates and its possible impact in WT. Further research is needed to understand if under the pressure of congestion, gender bias is exacerbated"

Reviewer 2

1. *Abstract: The abstract needs restructuring in the following order: background, objectives, methods, findings and conclusion along with policy implications. The abstract is lacking policy implications. For this purpose, a couple of sentences may be added to the last of abstract.*

   Following your suggestions we have restructured the abstract accordingly.

2. *Introduction: Introduction section is well written. Sufficient literature is reviewed. However, the authors have mostly cited the gender-differences from the western world. From developing world, along with an Indian study already cited, cite some more studies, such as those from Panezai, S., Ahmad, M. M., & Saqib, S. E. (2017). Factors affecting access to primary health care services in Pakistan: a gender-based analysis. Development in practice, 27(6), 813-827 and Panezai, S., Ahmad, M. M., & e Saqib, S. (2020). A Gender-Based Assessment of Utilization of Primary Health Care Services and Associated Factors in Pakistan. Ponte Journal, 76(1/1). Citing these studies, will help compare gender-differences from both the developing and developed word perspectives.*

   We agree with you that the paper by Panezai et.al (2020) is relevant to our work, and it has been included as follows: "In [12] the authors study gender-based utilization factors of primary health centers in Pakistan, finding statistical differences in predisposing, enabling and need factors."
   [12] Panezai, S., Ahmad, M. M., & e Saqib, S. (2020). A Gender-Based Assessment of Utilization of Primary Health Care Services and Associated

Factors in Pakistan. Ponte Journal, 76(1/1).

3. *Methods: Methods section is sufficiently explained. Designed elaborated and data described. The author(s) should give full names to the abbreviations such as FONASA and GES at first use.*

   Following your suggestion, we have included the full names for both FONASA and GES upon their first use.

4. *Results: Data is well presented and sufficiently explained.*

   Thank you very much. We notice that in the new manuscript, this section has been upgraded to include the results of the multilevel regression model.

5. *Discussion: The discussion section is lacking comparative analysis of current findings with those of existing studies. In this section, the data presented need to be compared with the existing literature and efforts should be put to find similarities and differences if any. The authors should try to convince the policy makers that that how meaningful are these differences? Lastly, this study has not mentioned clearly the potential limitations, the authors must document the limitations of the current study.*

   Materials and Methods, Results, and Discussion sections have substantially changed in the new version of the manuscript. We hope this new version is more complete.

   We included the following paragraph for limitations: "The main limitation of this study is that we did not explicitly consider the congestion of the healthcare system. Including the latter would be useful to understand compliance rates and its possible impact in WT. Further research is needed to understand if under the pressure of congestion, gender bias is exacerbated"

6. *Conclusion: The conclusion section fails to stipulate policy implications of the current study. The authors should come up with concrete policy level suggestions for addressing the gender differences.*

   We have enhance the conclusion including more concrete policy suggestions:

   "Our analysis shows that the existence of explicit opportunity guarantees for GES–OG does not prevent bias when considering the timeliness of treatment between women and men. This bias is impacted by the difference among providers and health districts, along with other observable patients' characteristics such as age and insurance holder status. We believe, with the limited evidence at hand, that differences in waiting times are most likely a product of a complex combination of several factors, where the role of women in society might be a fundamental component. Understanding these factors is part of our ongoing research. Once the reasons behind these biases are known, more specific, differentiated, gender oriented policies should be implemented. In the meantime, positive actions that facilitate timely treatment for women should be considered,

especially for those between 40 and 54 years old."

We thank the reviewers and associate editor for their input, which significantly improved the quality of this manuscript, and hope that our new improved version of the paper addresses and clarifies all their questions and comments.

Sincerely,

Susana Mondschein
María José Quinteros
Natalia Yankovic
