# Supplementary material for: Gender bias in the Chilean public health system: Do we all wait the same?
Source: PLoS One. 2020 Sep 24;15(9):e0239445. doi: 10.1371/journal.pone.0239445 (PMC7515657; doi:10.1371/journal.pone.0239445)
Supplement: Attachment — Submitted filename: Answer referee PLOS 2.pdf [file pone.0239445.s003.pdf]

July 17, 2020

Editor PLOS ONE

Dear Academic Editor and Reviewers,

Thank you very much for the time you have devoted to reading our paper "Bias in the Chilean public health system: Do we all wait the same?" (PONE-D-20-03609). In what follows, we answer in detail the reviewer's comments.

Reviewer 1

1. *Dear Authors, congratulations, you have made a great improvement in this revised manuscript. I really enjoyed reading it and appreciate what you have done.*

   We appreciate your positive feedback and the insightful comments you have provided in your reports. Our paper became a better one because of that.

2. *Let me just point to one more issue. I think you could do more to make your tables and figures more appealing. I mean, they could be more self-standing and comprehensible without referring to text.*

   Following your advice, we have improved the titles of tables and included notes to make them more self-standing.

3. *Therefore, I think you could explain all the abbreviations also in tables'/figures' 'Notes:' (at least at the first time they are used in tables/figures), see for example 'GES' in T1, 'GES-OG' in T2. Also, in my opinion, the abbreviations in the titles are not recommended; your title in T5 could be much more informative and give more details on what the numbers below show. For Tables 6 and 7, again in the 'Notes:' under the tables I think you could provide a brief explanation of how to interpret the numbers; what does negative/positive value mean. In figure 1, you do not have axis labels. In figure 2, the term 'Age group [5n,5n+4]' is not clear for me.*

   Following your suggestion, we have eliminated all abbreviations from the titles of tables and figures and added the explanation of any abbreviation at the bottom of figures/tables. We have changed the title of Table 5, including further details on what the numbers show. For Tables 6 and 7 we have included a note with a brief interpretation of the main findings. We have labeled the axis of Figure 1, and we have also improved the label of the group age in Figure 2.

4. *Generally, try to think about presenting tables and figures in the way that*

allows to find out as much as possible from tables and figures solely, with not much reference to the text. Notes under the tables/figures might be very useful for this purpose.

Tables and figures have been improved, making them easier to understand without referring to the main text.

Reviewer 2

1. *The authors have done good job and have incorporated all the suggestions of reviewer. This manuscript should be accepted for publication now.*

   We thank the reviewer for the positive feedback.

We thank the reviewers and associate editor for their input, which significantly improved the quality of this manuscript, and hope that this revised version of the paper addresses all their comments.

Sincerely,

Susana Mondschein
María José Quinteros
Natalia Yankovic
