# Supplementary material for: Gender bias in the Chilean public health system: Do we all wait the same?
Source: PLoS One. 2020 Sep 24;15(9):e0239445. doi: 10.1371/journal.pone.0239445 (PMC7515657; doi:10.1371/journal.pone.0239445)
Supplement: Attachment — Submitted filename: Reviewer_PONE-D-20-03609.docx [file pone.0239445.s001.docx]

**Reviewer’s comments**

This is very interesting study and may have significant policy implications for addressing gender differences in access to healthcare services in the Chilean public health system. However, there are some short comings which need to be addressed before it is considered for publication.

**Abstract:** The abstract needs restructuring in the following order: background, objectives, methods, findings and conclusion along with policy implications. The abstract is lacking policy implications. For this purpose, a couple of sentences may be added to the last of abstract.

**Introduction:**

Introduction section is well written. Sufficient literature is reviewed. However, the authors have mostly cited the gender-differences from the western world. From developing world, along with an Indian study already cited, cite some more studies, such as those from Panezai, S., Ahmad, M. M., & Saqib, S. E. (2017). Factors affecting access to primary health care services in Pakistan: a gender-based analysis. *Development in practice*, *27*(6), 813-827 and Panezai, S., Ahmad, M. M., & e Saqib, S. (2020). A Gender-Based Assessment of Utilization of Primary Health Care Services and Associated Factors in Pakistan. Ponte Journal, 76(1/1). Citing these studies, will help compare gender-differences from both the developing and developed word perspectives.

**Methods:** Methods section is sufficiently explained. Designed elaborated and data described. The author(s) should give full names to the abbreviations such as FONASA and GES at first use.

**Results:** Data is well presented and sufficiently explained.

**Discussion:** The discussion section is lacking comparative analysis of current findings with those of existing studies. In this section, the data presented need to be compared with the existing literature and efforts should be put to find similarities and differences if any. The authors should try to convince the policy makers that that how meaningful are these differences? Lastly, this study has not mentioned clearly the potential limitations, the authors must document the limitations of the current study.

**Conclusion:** The conclusion section fails to stipulate policy implications of the current study. The authors should come up with concrete policy level suggestions for addressing the gender differences.

Overall: This manuscript has the potential to be published provided that the above mentioned issues are addressed.
